# Socioeconomic conditions of elderly people in Kosovo: a cross-sectional study

**DOI:** 10.1186/1471-2458-12-512

**Published:** 2012-07-09

**Authors:** Naim Jerliu, Ervin Toçi, Genc Burazeri, Naser Ramadani, Helmut Brand

**Affiliations:** 1 Department of International Health, School for Public Health and Primary Care (CAPHRI), Faculty of Health, Medicine and Life Sciences, Maastricht University, Maastricht, The Netherlands; 2 Institute of Public Health, Pristine, Kosovo; 3 Institute of Public Health, Tirana, Albania

**Keywords:** Ageing, Education, Elderly people, Income, Kosovo, Poverty, Socioeconomic characteristics

## Abstract

**Background:**

Kosovo is the newest state in Europe facing a particularly difficult socioeconomic and political transition. The available evidence on socioeconomic conditions and quality of life of elderly people in Kosovo is scarce notwithstanding the ageing trend due to lowering of fertility rates and a higher life-expectancy. In this context, the aim of our study was to assess the socioeconomic conditions of elderly people in post-war Kosovo.

**Methods:**

A cross-sectional study was conducted in Kosovo in January-March 2011 including an age- sex-and residence (urban vs. rural)-stratified sample of 1,890 individuals (83.5% response) aged 65 years and over. A structured questionnaire included assessment of socio-demographic and socioeconomic characteristics including educational level and self-perceived poverty. Binary logistic regression was used to assess the association of self-perceived poverty with socio-demographic and socioeconomic factors.

**Results:**

The educational level in this representative sample of elderly people in Kosovo was quite low, particularly among women. About 47% of respondents perceived themselves as poor, or extremely poor (41% of men and 52% of women). In multivariable-adjusted models, self-perceived poverty was higher among older women, low educated individuals, urban residents, and elderly individuals living alone.

**Conclusions:**

Findings from this study indicate that the socioeconomic situation of the elderly population in Kosovo is rather challenging. Demographic trends coupled with the economic and political transition raise serious concerns about increasing needs for socioeconomic support of elderly people in Kosovo. Specific policies and actions should be considered by a number of stakeholders, including government and civil society in transitional Kosovo.

## Background

Steady increases in life expectancy coupled with reductions in fertility rates point to the great concern related to aging as a global societal concern worldwide [1,2]. Furthermore, such demographic trends imply important social changes and intensification of health care demands which will be increasingly difficult to accommodate in the context of limited resources [3]. Despite the growing body of evidence supporting the changes in demographic trends, little is known in terms of quality of life and social participation of elderly people [4].

It is important to gear scientific research towards aging because older people comprise a considerably large vulnerable group of society [5]: they may be more likely to live in poor quality housing including worse-off neighbourhoods; maybe more prone to acute illness and discrimination regarding the access to health care, and; are more financially dependent on social schemes or transfers from other people [6]. As a matter of fact, evidence indicates that elderly people aged 65 and over are at greater risk of poverty compared to general population [7]. Kosovo is the newest country in Europe consisting of the youngest European population. Expected life expectancy in Kosovo in 2008 was 67years for males and 71years for females [8]. Nevertheless, Kosovo unavoidably resembles the global trend of aging reflected by a considerable slowdown of population increase rate from 27% in 1981 to 9% in 2007 [9], indicating growing proportions of the older age groups over the years. Available data indicate that from 2003 to 2009 the proportion of persons aged ≤15years decreased by 5% (from 33.1% to 28.2%, respectively), whereas the proportion of individuals aged 65+ increased from 6.4% to 7%. The ageing effect could be attributed to lowering of fertility rates, a higher life-expectancy and emigration of working-age adults [8].

In Kosovo, the support for persons aged 65+ years is regulated through legislative measures. Nevertheless, current national programs, strategies and macro-policies do not sufficiently and adequately address health needs and socioeconomic challenges related to elderly people. The highly polarized political life and extensive reforms have overlooked the elderly population in many regards. According to a recent World Bank report, Kosovo is among the poorest countries in Europe [10] with 34% of population living below the national poverty line, of which 12% live in extreme poverty. Old people may constitute a large share of this proportion as the elderly segment of the population is considered to be at high risk of poverty [10,11]. Indeed, poverty in Kosovo may be of particular concern for the elderly population as suggested from a recent International Labour Organization report [9].

To date, however, there is lack of scientific evidence focused specifically on socioeconomic conditions of elderly people in Kosovo. In particular, poverty level among elderly people has not been properly assessed, which poses a serious obstacle for addressing correctly the unmet needs of this vulnerable subgroup of the population in Kosovo. In this context, given the rising trend of aging population coupled with a particularly unprepared socio-political and economic environment, we carried out a population-based survey aiming to assess the socioeconomic conditions of elderly people aged 65+ years in Kosovo, with a particular focus on self-perceived poverty levels.

## Methods

### Design and study population

A cross-sectional study was conducted in Kosovo in January-March 2011.

**Figure 1:**
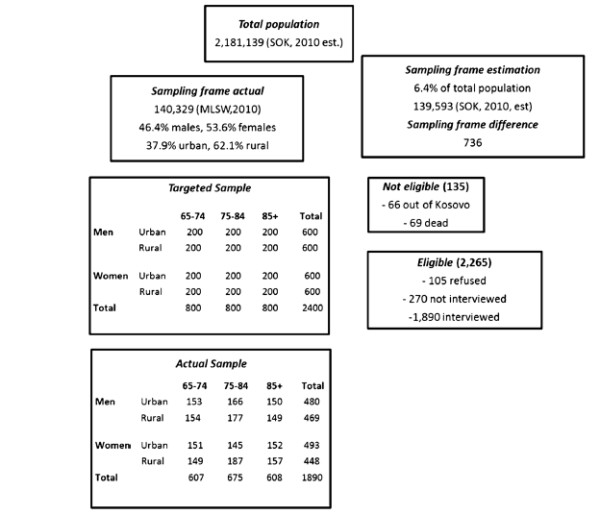
Sampling frame, sampling procedure and response rate of elderly people survey, Kosovo, 2011.

In 2010, the population of Kosovo was estimated at 2,181,139 and the segment aged 65+ years comprised 6.4% (139,593) of the overall population (Figure 1) [12]. According to the law of pension, entered into force in 2002, all individuals aged 65+ years benefit the *“basic old-age pension”* in the framework of a universal coverage policy. The actual data retrieved in 2010 from the Ministry of Labour and Social Welfare contained 140,329 individuals aged 65 or older registered as pension beneficiaries. This list was used as the sampling frame since all persons 65+ years are supposed to appear in this list including also people with disabilities and limitations. Based on this list, we drew an age- sex-and place of residence (urban vs. rural)-stratified sample of 2,400 individuals aged 65+ years. Twelve strata were established. Age-stratification consisted of three groups: 65–74, 75–84 and >85years. Each of the twelve strata consisted of a simple random sample of 200 individuals (Figure 1). The inclusion criteria were as follows: age 65+ years according to the list (sampling frame) and Kosovo citizenship.

Of the targeted 2,400 individuals aged 65 and over, 135 were not eligible (69 individuals were dead, whereas 66 persons had emigrated). Of the 2,265 eligible individuals, 270 participants could not be located, whereas other 105 individuals refused to participate. The final response rate was 83.5% (1,890/2,265) [Figure 1].

### Sample size calculation

Calculation of the sample size was made by use of WINPEPI [13] for a number of socioeconomic hypotheses such as educational level and self-perceived poverty. The assumed prevalence of self-perceived poverty was set at 50%, the significance level (two-tailed) at 5%, and the power of the study at 80%. Based on these conservative calculations, the required minimal sample size was about 1,500 individuals. We decided to sample 2400 individuals (200 for each of the 12 strata explained above) in order to increase the power of the study.

### Data collection

A structured interviewer-administered questionnaire was used tapping socio-demographic characteristics (age, sex, place of residence [urban vs. rural area], ethnicity [Albanian, Serbian, Roma and other] and religion [Muslim, Catholic and Orthodox]) and socioeconomic factors (education [years of formal schooling – in the analysis categorized into 0, 1–8 and >9years], living arrangements [not alone vs. alone], family finances at the end of the month (enough vs. not enough to make ends meet) and self-perceived poverty level [upon question: *“How do you perceive your poverty level”* – measured on a scale ranging from 1 (extremely poor) to 5 (fair); in the analysis, self-perceived poverty was dichotomized into poor (1–3) vs. not poor (4–5)].

The survey was approved by the Professional Ethical Board of the Ministry of Health of Kosovo. All individuals who agreed to participate gave their informed consent after being explained the aim and procedures of the survey.

### Statistical analysis

Socio-demographic and socioeconomic sample estimates were standardized for age, sex and place of residence in accordance with the respective strata from the sampling frame (Figure 1). Absolute numbers and their respective percentages from the study sample, and standardized (population-weighted) percentages with their respective 95% confidence intervals (95% CIs) were reported.

Age-adjusted binary logistic regression was used to assess the association of self-perceived poverty (not poor vs. poor) with socio-demographic characteristics. Multivariable-adjusted binary logistic regression, applying a backward stepwise procedure with a P-value to exit >0.10, was used to assess the independent association of self-perceived poverty with covariates (age, sex, education, residence, marital status and living arrangements – ethnicity and religion were excluded from further consideration since they showed almost no discriminatory power). Age-adjusted and multivariable-adjusted odds ratios (ORs) and their respective 95% CIs were calculated. Hosmer-Lemeshow test was used to assess the fit of the models; all models met the criteria [14]. Statistical Package for Social Sciences, version 15.0, Chicago, Illinois was used for all the analyses.

## Results

Socio-demographic characteristics of elderly people aged 65years and over included in this survey are presented in Table 1. Mean age of study participants (overall: 73.4years) was similar between men and women (72.99 ± 5.9 vs. 73.7 ± 6.6years, respectively). About two thirds of male and female respondents resided in rural areas (overall: 62.1%). On average, respondents had 4.5years of formal education with considerable differences between men and women (mean years of formal education: 6.6 ± 4.6 in men 2.7 ± 3.2 in women). Almost half of the women (47.9%) had no formal education compared to 16.7% of men, whereas the inverse trend was observed for 9 or more years of formal education (2.5% of women vs. 23.2% of men had 9+ years of formal education, respectively). About three quarters of men (71.1%) reported to be married at the time of the survey compared to about 40% of women. The reverse trend was observed regarding the proportions of widowed (26.8% of men vs. 56.7% of women). The sample was quite homogenous in terms of ethnicity or religion with 90% being Albanians and 91% of Muslim affiliation, respectively (Table 1). As a matter of fact, ethnicity and religion did not reveal any discriminatory power as they were equally distributed among the socio-economic groups in the study sample; therefore, given also the small numbers in the non-Albanian and non-Muslim categories, these variables were excluded from further analysis.

**Table 1:** Socio-demographic characteristics of a population-based sample of elderly people in Kosovo, 2011

**Numerical variables**	**Men (n = 949)**		**Women (n = 941)**		**Total (n=1890)**	
	Sample mean ± SD^*^	**Standardized mean (95% CI)**^**†**^	**Sample mean ± SD**	**Standardized mean (95% CI)**	**Sample mean ± SD**	**Standardized mean (95% CI)**
**Age *(years)***	72.99 ± 5.94	72.99 (72.94-73.03)	73.67 ± 6.59	73.67 (73.62-73.72)	73.35 ± 6.31	73.35 (73.32-73.38)
**Education *(years)***	5.58 ± 4.86	6.58 (6.54-6.62)	1.91 ± 2.92	2.70 (2.68-2.72)	3.76 ± 4.41	4.51 (4.48-4.53)
**Categorical variables**	**Men (n = 949)**		**Women (n = 941)**		**Total (n = 1890)**	
	Sample N (%)^*^	**Standardized% (95% CI)**^**†**^	**Sample N (%)**	**Standardized% (95% CI)**	**Sample N (%)**	**Standardized% (95% CI)**
**Age group:**						
65–74	307 (32.3)	67.0 (66.60-67.32)	300 (31.9)	63.1 (62.71-63.40)	607 (32.1)	64.9 (64.62-65.12)
75–84	343 (36.1)	29.4 (29.08-29.78)	332 (35.3)	31.2 (30.85-61.52)	675 (35.7)	30.4 (30.13-30.61)
85+	299 (31.5)	3.6 (3.47-3.76)	309 (32.8)	5.8 (5.54-5.98)	608 (32.2)	4.8 (4.65-4.88)
**Residence:**						
Urban	469 (49.4)	38.7 (38.36-39.11)	448 (47.6)	37.2 (36.85-37.54)	917 (48.5)	37.9 (37.66-38.16)
Rural	480 (50.6)	61.3 (60.89-61.64)	493 (52.4)	62.8 (62.46-63.15)	973 (51.5)	62.1 (61.84-62.34)
**Ethnicity:**						
Albanian	857 (90.3)	91.1 (90.87-91.28)	843 (89.6)	89.0 (88.76-89.18)	1700 (89.9)	89.9 (89.79-90.10)
Serbian	57 (6.0)	5.5 (5.31-5.66)	63 (6.7)	7.2 (7.05-7.42)	120 (6.3)	6.4 (6.29-6.55)
Roma	9 (1.0)	0.5 (0.43-0.54)	3 (0.3)	0.2 (0.21-0.29)	12 (0.6)	0.4 (0.33-0.39)
Other	26 (2.7)	2.9 (2.81-3.07)	32 (3.4)	3.6 (3.43-3.70)	58 (3.1)	3.3 (3.18-3.37)
**Religion:**						
Muslim	868 (91.5)	92.4 (92.21-92.62)	858 (91.2)	90.2 (90.00-90.43)	1726 (91.3)	91.2 (91.09-91.38)
Catholic	24 (2.5)	2.1 (1.99-2.22)	20 (2.1)	2.6 (2.44-2.67)	44 (2.3)	2.3 (2.26-2.42)
Orthodox	57 (6.0)	5.5 (5.31-5.66)	63 (6.7)	7.2 (7.05-7.42)	120 (6.4)	6.4 (6.29-6.55)
**Education:**						
0 years	251 (26.4)	16.7 (16.39-16.96)	585 (62.2)	47.9 (47.55-48.26)	836 (44.2)	33.4 (33.16-33.66)
1–8 years	507 (53.4)	59.6 (59.25-60.00)	325 (34.5)	48.3 (47.93-48.65)	832 (44.0)	53.6 (53.29-53.81)
9+ years	186 (19.6)	23.2 (22.79-23.65)	18 (1.9)	2.5 (2.36-2.58)	204 (10.8)	12.1 (11.93-12.27)
**Marital status:**						
Married	554 (58.3)	71.1 (70.73-71.43)	243 (25.8)	39.1 (38.77-39.41)	797 (42.2)	54.0 (53.69-54.21)
Single	10 (1.1)	0.5 (0.43-0.54)	11 (1.2)	1.7 (1.61-1.80)	21 (1.1)	1.1 (1.08-1.20)
Separated	5 (0.5)	0.4 (0.33-0.42)	9 (1.0)	1.0 (0.94-1.08)	14 (0.7)	0.7 (0.67-0.76)
Widowed	366 (38.6)	26.8 (26.44-27.12)	663 (70.4)	56.7 (56.38-57.09)	1029 (54.4)	42.8 (42.57-43.09)
**Living arrangements:**						
Living alone	57 (6.0)	5.0 (4.86-5.19)	58 (6.2)	5.1 (4.97-5.28)	115 (6.1)	5.1 (4.96-5.19)
Not living alone	892 (94.0)	95.0 (94.81-95.14)	882 (93.7)	94.7 (94.55-94.88)	1774 (93.9)	94.8 (94.72-94.95)

^*^ Mean values (and their respective standard deviations) and absolute numbers (and their respective column percentages) in the actual study sample. Discrepancies in the totals for educational level, marital status and living arrangements are due to missing covariate values.

^**†**^Age-sex and-residence standardized mean values and column percentages and their respective 95% confidence intervals (95%CIs).

About half of respondents (46.9%) perceived themselves as ‘poor’ or ‘extremely poor’ (40.8% of men vs. 52.0% of women) (Table 2).

**Table 2:** Socioeconomic characteristics of the study population

**Socioeconomic characteristic**	**Men (n = 949)**		**Women (n = 941)**		**Total (n = 1890)**	
	Sample N (%)^*^	**Standardized% (95% CI)**^**†**^	Sample N (%)^*^	**Standardized% (95% CI)**	Sample N (%)^*^	**Standardized% (95% CI)**
**Family finances at end of month:**						
Not enough	667 (70.3)	67.6 (67.20-67.92)	718 (76.3)	76.5 (76.23-76.83)	1385 (72.4)	72.4 (72.13-72.60)
Enough	275 (29.0)	31.5 (31.11-31.83)	215 (22.8)	22.7 (22.42-23.02)	490 (26.8)	26.8 (26.55-27.01)
*Missing*	7 (0.7)	1.0 (0.90-1.05)	8 (0.9)	0.8 (0.69-0.82)	15 (0.9)	0.9 (0.81-0.91)
**Self-perceived poverty:**						
Extremely poor	121 (12.8)	13.2 (12.96-13.44)	172 (18.3)	18.5 (18.27-18.82)	293 (15.5)	16.1 (15.88-16.26)
Poor	308 (32.4)	27.6 (27.22-27.91)	329 (35.0)	33.5 (33.18-33.86)	637(33.7)	30.8 (30.51-31.00)
Moderate	346 (36.5)	39.5 (39.08-39.84)	296 (31.4)	33.0 (32.63-33.31)	642 (34.0)	36.0 (35.73-36.23)
Not poor	106 (11.2)	12.4 (12.14-12.65)	83 (8.8)	8.8 (8.63-9.04)	189 (10.0)	10.5 (10.33-10.65)
Fair	44 (4.6)	4.8 (4.68-5.01)	45 (4.8)	4.5 (4.39-4.69)	89 (4.7)	4.7 (4.57-4.79)
*Missing*	24 (2.5)	2.5 (2.41-2.65)	16 (1.7)	1.6 (1.52-1.70)	40 (2.1)	2.0 (1.96-2.11)

^*^ Mean values (and their respective standard deviations) and absolute numbers (and their respective column percentages) in the actual study sample.

^**†**^Age-sex and-residence standardized mean values and column percentages and their respective 95% confidence intervals (95%CIs).

In age-adjusted comparisons (Table 3), women were significantly more likely to perceive themselves as poor compared to men (OR=1.4, 95% CI=1.1-1.6). Same finding was evident for urban residents compared to their rural counterparts (OR=1.3, 95% CI=1.1-1.5). Individuals aged 85+ were significantly more likely to perceive themselves as poor compared to the youngest age-group (OR=1.3, 95% CI=1.0-1.6). Compared to the highly educated individuals (9 or more years of formal education), those without formal education were significantly more likely to perceive themselves as poor (OR=1.4, 95% CI=1.0-2.0). Widowed individuals were significantly more likely to perceive themselves as poor compared to their married counterparts (OR=1.3, 95% CI=1.1-1.6), whereas participants who reported to be single were two times more likely to perceive themselves as poor compared to the married ones (OR=2.7, 95% CI=1.0-7.0). Individuals living alone were more likely to perceive themselves as poor compared to those not living alone (OR=2.5, 95% CI=1.6-3.7).

**Table 3:** Association of socio-demographic and socioeconomic characteristics with self-perceived poverty; age-adjusted analysis

**Variable**	OR^*^	95% CI^*^	**P**
**Sex:**			
Men	1.00	Reference	0.001
Women	1.36	1.13-1.63	
**Age group:**			**0.072 (2)**^**†**^
65–74 years	1.00	Reference	-
75–84 years	1.13	0.90-1.41	0.285
85+ years	1.31	1.04-1.64	0.022
**Residence:**			
Rural	1.00	Reference	0.012
Urban	1.27	1.06-1.53	
**Educational level:**			**0.097 (2)**
9+ years	1.00	Reference	-
1–8 years	1.34	0.98-1.82	0.070
0 years	1.43	1.03-1.99	0.031
**Marital status:**			**0.029 (3)**
Married	1.00	Reference	-
Single	2.66	1.01-7.01	0.047
Separated	1.14	0.39-3.27	0.814
Widowed	1.29	1.06-1.58	0.013
**Living arrangements:**			
Not living alone	1.00	Reference	
Living alone	2.47	1.63-3.74	<0.001

^*^ Age-adjusted odds ratios (ORs: poor vs. not poor) and 95% confidence intervals (CIs) from binary logistic regression.

^**†**^ Overall p-value and degrees of freedom (in parentheses).

After adjustment for all covariates (Table 4) using a backward stepwise procedure, lack of formal education and/or 1–8years of formal schooling were both associated with increased likelihood of self-perceived poverty (OR=1.6, 95% CI=1.1-2.3 and OR=1.4, 95% CI=1.0-2.0, respectively). Furthermore, female gender and urban residence remained significant predictors of self-perceived poverty (OR=1.3, 95% CI=1.0-1. 6 and OR=1.4, 95% CI=1.2-1.7, respectively).

**Table 4:** Association of socio-demographic and socioeconomic characteristics with self-perceived poverty; multivariable-adjusted analysis

**Variable**	OR^*^	95% CI^*^	**P**
**Sex:**			
Men	1.00	Reference	0.024
Women	1.27	1.03-1.56	
**Age group:**			
65–74 years			
75–84 years			
85+ years			
**Residence:**			
Rural	1.00	Reference	<0.001
Urban	1.42	1.17-1.73	
**Educational level:**			**0.037 (2)**^**†**^
9+ years	1.00	Reference	-
1–8 years	1.41	1.01-1.96	0.044
0 years	1.60	1.12-2.28	0.010
**Marital status:**			
Married			
Single			
Separated			
Widowed			
**Living arrangements:**			
Not living alone	1.00	Reference	<0.001
Living alone	2.66	1.73-4.07	

^*^ Multivariable-adjusted odds ratios (ORs: poor vs. not poor) and 95% confidence intervals (CIs) from binary logistic regression. All variables presented in the table were included in a backward stepwise elimination procedure with a p-value to exit set at >0.10. Empty cells refer to the variables excluded from the model.

^**†**^ Overall p-value and degrees of freedom (in parentheses).

## Discussion

The main findings of this survey, which included a nationwide representative sample of elderly people aged 65years and over in Kosovo, consist of a high rate coupled with a significant difference in men vs. women regarding the educational attainment and self-perceived poverty levels. Our results indicate a significant inverse association of self-perceived poverty with education, but a positive relationship with female gender, urban residence and living alone.

World population (including the Balkan countries) is ageing and the proportion of individuals aged 65+ years will be quickly expanding in the upcoming years, notwithstanding the decline in population figures in several countries [2,3,15]. Kosovo remains the youngest country in Europe and reports the lowest share of individuals aged 65+ compared to other countries in the region: the share of 65+ population is about 10% in Albania, whereas in Bulgaria, Croatia, Latvia and Poland it is around 17% [16].

Our findings regarding demographic characteristics are comparable to those reported previously in Kosovo and in other countries of the region. The 2009 Demographic, Social and Reproductive Health Survey in Kosovo reported similar findings with our study, although the sample was not restricted to elderly people [8]. On the other hand, it is consistently reported that the proportion of elderly people living in rural areas is higher than those living in urban areas and more women are found in the oldest age groups compared to men [1] and, similar to the world statistics, the majority of elderly men are not widowed, whereas the majority of elderly women are widowed [1,17]. This is important since not being widowed affects positively the well-being of elderly people and their living arrangements [18]. In age-adjusted analysis, we obtained evidence that individuals being single were almost three times more likely to perceive themselves as poor compared to their married counterparts.

Our survey revealed that only 5% of elderly people were living alone and a similar situation is present in developing countries in general [1]. Conversely, figures from the neighbouring Albania and Serbia suggest that the proportion of elderly individuals living alone is 20% [19,20], whereas in Turkey it is around 30% [17]. The 2007 World Economic and Social Survey reported that 25% of individuals aged 60+ in developed countries and 7% in developing countries live alone [21]. Similar to other studies [21-23], we found that living alone is a risk factor for feeling poor among the elderly: in multivariable-adjusted analysis, individuals aged 65+ who were living alone were 2.5 times more likely to perceive themselves as poor compared to those not living alone.

On the other hand, the distribution of ethnicity and religiosity in our elderly sample was similar to other sources of information in the Republic of Kosovo (8), with an overwhelming majority of Albanians (90%) [Muslims: 91.2%; Orthodox: 6.4%].

The gender educational gap, somewhat evidenced in previous surveys in Kosovo [8], was nevertheless quite striking in our study. Our findings differ slightly from those reported by the Demographic, Social and Reproductive Health Survey in Kosovo in 2009 which reported that 21.1% of males aged 65+ had no formal schooling compared to 64.4% of females of the same age [in our study, these figures were: 16.7% and 47.9%, respectively]. These differences could be due to the cohort effect: it is likely that younger cohorts exhibit higher educational levels due to a better access to formal schooling compared to the older generations. An earlier report in Kosovo suggested that, in 2005, about 49% of individuals aged 65+ had no formal education [5]. Another fact supporting the cohort effect is the decrease in illiteracy rates in Kosovo from 41.1% in 1961 to 18% in 1981 [24]. The low proportion of formal education in Kosovo and male/female differences regarding formal schooling have been suggested to be rooted in political, social and economic context of the country since the middle of the 20^th^ Century [24]. The low rates of formal schooling are not unique for Kosovo: in Turkey, in 2007, 84% of elderly women and 70% of elderly men (aged 60+) did not receive any education at all or had dropped out from primary school [17].

In order to understand the financial situation of elderly people in Kosovo, social policies surrounding elderly people in this country should be considered. According to the Law on Pensions (2001), the pension scheme in Kosovo is based in the so-called “three pillar system”. The first pillar states that every person aged 65+ in Kosovo is entitled to a basic pension, which is the same for all the elderly and aims to reduce the poverty. Virtually, all persons aged 65+ are enrolled in this system. However, the amount of money the elderly receive is very small and not sufficient to meet their needs. In 2010, the amount of basic pension was 45 Euro/month [25]. The second pillar of financing of elderly is the savings pension. In the framework of high unemployment rates and black labour, the proportion of elderly who benefit from this social scheme is very limited. The third pillar is a voluntary supplemental system [9,26].

Finally, there are social and family services offered for elderly people who are unable to look after themselves, which are provided by governmental and non-governmental organizations. A person living on social assistance in Kosovo receives on average 0.46 Euro/day [27]. However, according to UNDP reports, about 25% of families in Kosovo receive remittances from relatives abroad. On average, each of these families receives 2,136 Euro/year [28]. Therefore, notwithstanding the financial difficulties of elderly people in Kosovo, they also benefit from remittances of their close relatives who work abroad.

More women than men are unemployed [29], meaning that fewer women can benefit from savings pension compared to men, which partly explains the higher rate of self-perceived poverty among women. Furthermore, the pattern of lower school enrolment and school completion among women is present. The boosting factors for lower school attainment among women are probably cultural: early marriages and the public opinion about the traditional roles of women within the family [5]. The gender discrimination is present in Kosovo similar to other countries in the region, such as Turkey [17].

According to the Kosovo Health Law (2004), health care services are provided free of charge in public health institutions for the citizens over 65 years of age [30].

The situation regarding the impact of social support for elderly in Kosovo is similar to other countries in the region. There is evidence from numerous studies that participation in pension schemes is associated with a reduction of poverty among elderly or reducing the intensity of poverty, including rural area residents [21].

A considerable help for the elderly consist of financial sources other than governmental schemes. A survey among elderly aged 65+ in Albania reported that 66% of them receive some financial support from their family members or other sources such as renting properties [31]. In Turkey, elderly people had one or more financial sources for sustaining them, and more men were entitled to such incomes compared to women [17]. In developing countries, these informal support mechanisms are often not stable and not reliable sources of income thus providing only limited security for the elderly people [21].

Efforts to alleviate poverty among elderly people have proven not successful, at least in some countries [17]. The situation is similar in Kosovo.

Regarding the prevalence of poverty, our report suggests that 16.1% of people aged 65+ are extremely poor (13.2% of men and 18.5% of women) whereas the UN ageing report indicated that 13.3% of worldwide individuals aged 65+ are poor [1]. Another study in Kosovo reported that 17.1% of elderly aged 65+ are extremely poor [5]. Another study among elderly aged 65+ in Albania found that 16.8% of them considered themselves as extremely poor [31], whereas 24% of elderly aged 60+ in Turkey were reported to be poor [17]. In Serbia, 14.7% of persons aged 65+ were reported to be poor [20]; the poverty level was higher in women, the low educated (20.2%) and in rural residents (18.6%) [20]. Findings from our survey suggest that the proportion of individuals perceiving themselves as poor or very poor is higher in urban areas compared to rural areas. This finding is supported by other studies in Kosovo [5]. However, it is difficult to compare poverty levels since no standardized measurement methodology has been adopted.

In our study, there was evidence of a significant association between educational level and self-perceived poverty. Furthermore, individuals living alone reported higher rates of extreme poverty compared to those not living alone, a finding which has been consistently reported from other studies conducted elsewhere [21,32]. Commonly, women have fewer education opportunities and, therefore, fewer employment chances. Consequently, fewer women benefit from pension schemes and, ultimately, elderly women are at higher risk of poverty compared to men. Furthermore, women live longer and hence their chances of living alone are higher with all the consequences this bears in terms of poverty.

### Study limitations

Potential limitations of our study include the generalizability of the findings, differential reporting of socioeconomic characteristics, particularly of self-perceived poverty level, and the study design.

We employed an age- sex-and residence-stratified sample; the stratum sampling may affect the distribution of socioeconomic characteristics among elderly people. However, all socio-demographic and socioeconomic estimates were standardized for age, sex, and place of residence to account for the stratification approach employed in the sampling procedure.

Although we cannot exclude the possibility of reporting bias, there seems no convincing reason for elderly people’s categories differing in age, sex, place of residence and educational level to have reported differently about their self-perceived poverty levels.

Furthermore, the findings of our study should be interpreted with caution, as the observed relationships from cross-sectional studies are not assumed to be causal.

## Conclusion

In summary, the socioeconomic situation of the elderly population in Kosovo seems rather challenging. The urbanization process and the internal and external migration increase the chances of elderly individuals for living alone. In addition, lack of formal education, especially among women, and the hectic context of a transitional society should be considered as major marginalizing factors which exacerbate poverty levels among elderly people in the emerging state of Kosovo.

In conclusion, demographic trends coupled with a society in economic and political transition raise serious concerns about increasing needs for socioeconomic support and social inclusion of elderly people in Kosovo. There is a low level of societal preparation in Kosovo capable to deal with socioeconomic needs of elderly people. Therefore, specific policies and actions should be considered by a number of stakeholders, including government and civil society in transitional Kosovo.

## Competing interests

The authors declare that they have no competing interests.

## Authors’ contribution

NJ contributed to the study conceptualization and design, acquisition of the data, analysis and interpretation of the data and writing of the article. ET, GB and HB contributed to the study conceptualization and design, analysis and interpretation of the data and writing of the article. NR commented on the manuscript. All authors have read and approved the submitted manuscript.

## Pre-publication history

The pre-publication history for this paper can be accessed here:

http://www.biomedcentral.com/1471-2458/12/512/prepub
